# Efficacy of Mesenchymal Stem Cell Therapy for Steroid-Refractory Acute Graft-Versus-Host Disease following Allogeneic Hematopoietic Stem Cell Transplantation: A Systematic Review and Meta-Analysis

**DOI:** 10.1371/journal.pone.0136991

**Published:** 2015-08-31

**Authors:** Xiaomei Chen, Chunyan Wang, Jin Yin, Jinhuan Xu, Jia Wei, Yicheng Zhang

**Affiliations:** Department of Hematology, Tongji Hospital, Tongji Medical College, Huazhong University of Science and Technology, Wuhan, China; University Hospital of Salamanca, SPAIN

## Abstract

**Background:**

Mesenchymal stem cells (MSCs) have been broadly used experimentally in various clinical contexts. The addition of MSCs to initial steroid therapy for acute graft-versus-host disease (aGVHD) may improve patient outcomes. However, investigations regarding prognostic factors affecting the efficacy of MSC therapy for steroid-refractory aGVHD remain controversial. We thus conducted a systematic review and meta-analysis of published clinical trials to determine possible prognostic factors affecting the efficacy of MSCs in treating steroid-refractory aGVHD.

**Methods and Findings:**

Clinical trials using MSC therapy for steroid-refractory aGVHD were identified by searching PubMed and EMBASE databases. A total of 6,963 citations were reviewed, and 13 studies met the inclusion criteria. A total of 301 patients from thirteen studies were included. Of these, 136 patients showed a complete response (CR), and 69 patients displayed a partial (PR) or mixed response (MR). In total, 205 patients exhibited overall response (ORR). Patients with skin steroid-refractory aGVHD showed a better clinical response than gastrointestinal (CR: odds ratio [OR] = 1.93, 95% confidence interval [95%CI]: 1.05–3.57, p < 0.05) and liver (CR: OR = 2.30, 95%CI: 1.12–4.69, p < 0.05, and ORR: OR = 2.93, 95%CI: 1.06–8.08, p < 0.05) steroid-refractory aGVHD. Those with grade II steroid-refractory aGVHD exhibited a better clinical response following MSC therapy than recipients with grade III–IV (CR: OR = 3.22, 95%CI: 1.24–8.34, p < 0.05). Completion therapy may improve the CR but reduce ORR compared with induction therapy (CR: OR = 0.20, 95%CI: 0.09–0.44, p < 0.05; ORR: OR = 2.18, 95%CI: 1.17–4.05, p = 0.01). There was also a trend towards a better clinical response in children compared with adults (CR: OR = 2.41, 95%CI: 1.01–5.73, p = 0.05).

**Conclusions:**

Age, skin involvement, lower aGVHD grade, and the number of infusions are the main prognostic factors affecting the efficacy of MSC therapy for steroid-refractory aGVHD.

## Introduction

Allogeneic hematopoietic-stem-cell transplantation (allo-HSCT) is the treatment of choice for many malignant and non-malignant hematological diseases [1]. Following allo-HSCT, acute graft-versus-host disease (aGVHD) is a life-threatening complication associated with HLA mismatch, high recipient age, low marrow cell dose, and splenectomy [2, 3]. Steroid is the initial treatment for controlling aGVHD; however, in 30–50% of patients, aGVHD is not controlled with first-line steroid therapy and requires further therapeutic intervention [4]. In a retrospective analysis of 864 patients with aGVHD [5], patients who failed to respond to therapy at day 28 after initial treatment were 2.78 times more likely to experience higher treatment-related mortality compared with those who demonstrated a response. Thus, the outcomes for non-responders are poor, and those patients who failed to respond to the initial treatment warrant a better second-line therapy.

Although prophylaxis for aGVHD and primary therapy with steroid is well established, second-line therapy is uncertain. Second-line agents include anti-thymocyte globulin [6, 7], visilizumab [8], denileukin diftitox [9], daclizumab [10–12], infliximab [13, 14], etanercept [15], psoralen and ultraviolet light A therapy [16], extracorporeal photopheresis [17–19], mycophenolate mofetil [20, 21], sirolimus [22], and pentostatin [23]. Despite these second-line treatments, the prognosis for steroid-refractory aGVHD remains disappointing owing to a higher risk of infectious complications, immunosuppression-related toxicity, and incomplete GVHD remission [24, 25]. The development of a better treatment strategy for steroid-refractory aGVHD is important to improve long-term survival for allo-HSCT recipients.

Among the most recent therapeutic methods for steroid-refractory aGVHD, mesenchymal stem cells (MSCs) hold a relatively crucial position. These are multipotent progenitor cells that exhibit extensive immunomodulatory properties. They inhibit the T and B cell response by arresting them in the G0/G1 phase of the cell cycle, prevent the antigen-presenting function of monocytes, and increase regulatory T cell expansion. Additionally, MSCs are known to escape immune rejection, thus allowing their use in a human leukocyte antigen-mismatched setting [26, 27].

Based upon early results of MSCs for the treatment of steroid-refractory aGVHD and an encouraging safety profile, many studies have been conducted to determine whether the addition of MSCs to initial steroid therapy for aGVHD would improve patient outcomes. Most studies identified MSCs as a promising treatment for severe steroid-resistant aGVHD. However, investigations regarding factors affecting the efficacy of MSC therapy for steroid-refractory aGVHD remain controversial. Some data exhibited a better clinical response in skin steroid-refractory aGVHD [28–33], some hinted at a better clinical response in gastrointestinal (GI) steroid-refractory aGVHD [34], whereas others showed no difference in response [35]. In addition, it is also unclear whether children showed a better clinical response compared with adults, whether fewer organs involved indicated a better clinical response than more organs involved—for example, some recipients only had skin steroid-refractory aGVHD while others may suffer from skin, GI, and liver steroid-refractory aGVHD, whether grade II steroid-refractory aGVHD showed a better clinical response than grade III–IV steroid-refractory aGVHD, or whether additional infusion of MSCs can improve the clinical response. Each of these factors has exhibited controversy. To help provide clarification, we conducted a systematic review and meta-analysis. We propose that through confirmation of these relationships, we will provide doctors with better guidance and support planning for treatment in steroid-refractory aGVHD.

## Material and Methods

### Identification and inclusion criteria of relevant studies

A bibliographic search was performed on the PubMed and Embase databases for studies investigating factors affecting the efficacy of MSCs in steroid-refractory aGVHD; studies from January 1974 to March 2015 were included in the search. The search strategies were as follows: (MSC OR mesenchymal stem cell) AND (GVHD OR graft versus host disease); results were limited to human studies and those published in English. Manual searches of reference lists from potentially relevant papers were also performed to identify any additional studies that may have been missed in the database search.

### Inclusion criteria

Only data from full, published papers (no meeting or conference abstracts or letters or case reports) were used. Studies included in this meta-analysis had to (a) use MSCs for the treatment of refractory aGVHD, (b) report factors affecting the efficacy of MSCs for treating steroid-refractory aGVHD, and (c) be written in English. Review articles and duplicate publications were excluded. Each article was checked independently for inclusion and exclusion criteria by two investigators (Chen and Wang). All manuscripts were reviewed to obtain the most complete information possible.

### Data extraction

Two investigators (Chen and Wang) performed double-blinded data extraction and methodological quality assessment. Data extracted from these studies included the first author, published year, nation of corresponding author, number of patients, donor type, patient age, patient sex, source of stem cells, number of children and adults, underlying diseases, conditioning regimen, GVHD prophylaxis, aGVHD grade, number of MSC infusions, and dose of MSCs. Two investigators (Chen and Wang) performed this work independently and reached a consensus on all items.

### Definitions

Mixed response (MR) was defined as improvement in staging of one organ with no change in others; partial response (PR) was defined as a decrease in staging but no resolution of all signs; complete response (CR) included cases of resolution of all signs, and overall response (ORR) included CR, PR and MR [32]. The average dose administered and average frequency of administration of MSCs varied in different studies except for three included studies [29, 36, 37]. In their studies, patients received 8 biweekly intravenous infusions of 2×10^6^ hMSCs/kg for 4 weeks (induction therapy), with an additional 4 weekly infusions after day 28 for patients who achieved either a partial or mixed response. Therapy was completed when all needed MSCs infusions were finished (completion therapy).

### Statistical analyses

Odds ratios (ORs) and their corresponding 95% confidence intervals (95%CIs) were used to assess the strength of factors associated with the efficacy of MSC therapy for steroid-refractory aGVHD. Data were tested using the Mantel-Haenszel method with fixed or random effect models. A p-value less than 0.05 was considered statistically significant. All p values were obtained using a two-sided test. I^2^ tests were performed to quantify the degree of heterogeneity between studies. Heterogeneity was considered present when the p value of Cochran’s Q test was <0.10 and the I^2^ statistic was >50%. If significant heterogeneity existed, a random-effect model was used to pool the data; otherwise, a fixed-effect model was applied. Forest plots were used to summarize the results of included studies. Begg’s and Egger’s tests were used to examine publication bias. Two independent sample t-test was used to compare patients’ clinical response between studies using fetal bovine serum and studies using human platelet lysate as culture medium. We included studies regarding factors affecting patients’ clinical responses after receiving MSCs for steroid-refractory aGVHD. Several main factors were finally identified after full-text screening of all included articles. The pooled ORs with 95%CIs were performed for children compared to adults, one or two involved organs compared to three involved organs, one involved organ compared to two involved organs, skin compared to GI steroid-refractory aGVHD, skin involvement compared to liver involvement, GI involvement compared to liver involvement, grade II compared to grade III–IV, one infusion compared to more than one infusion and MSC induction therapy compared to completion therapy. We extracted both the complete response (CR) and overall response (ORR) as the endpoint of the clinical response. Analyses were performed using Review Manager software (v. 5.3, http://ims.cochrane.org/revman/download) and the SPSS software package (Version 19.0, SPSS Inc., Chicago, IL, USA).

## Results

### Literature search

**Fig 1:**
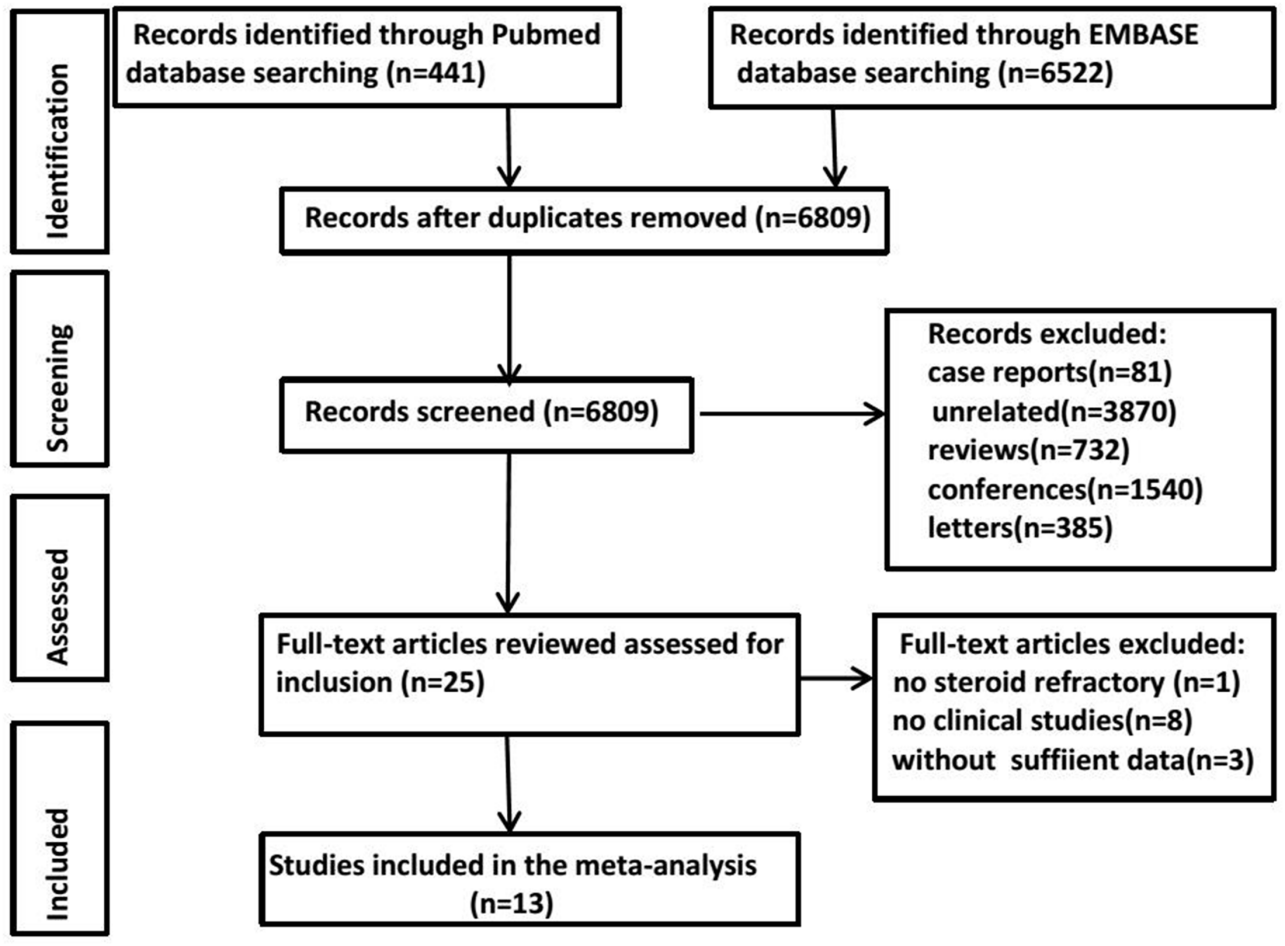
Flow diagram of the literature search process for identifying studies.

A systematic search of PubMed and EMBASE databases yielded 441 and 6522 results, respectively. After removing duplicates, the titles and abstracts from the remaining 6809 studies were screened, and 25 potentially eligible studies for the meta-analysis were retained. After retrieving the full-text version of the aforementioned 25 studies, one study was excluded because they used MSCs to treat aGVHD as a first-line therapy rather than steroid-refractory aGVHD. Four studies did not provide sufficient data. Eight studies were excluded because they were not clinical trials. The literature search process is summarized in Fig 1.

### Systematic review

Thirteen studies (eight prospective and five retrospective), including 333 patients, met all inclusion criteria and were included in the meta-analysis. Twenty-seven patients were diagnosed with chronic GVHD, and the data for five patients were unavailable, leaving a total of 301 patients that were included. Introna et al. [30] treated 15 children and 25 adults experiencing steroid-resistant grade II to IV GVHD, but only 12 children and 19 adults used MSCs for steroid-refractory aGVHD. Ringdén et al. [31] used MSCs to treat eight patients with grade III to IV steroid-refractory aGVHD and one with extensive chronic GVHD. Therefore, we only included the eight patients in this study. Herrmann et al. [33] undertook a phase I trial in 19 patients with steroid-refractory aGVHD and chronic GVHD using bone marrow-derived MSCs; only 12 patients were treated for steroid-refractory aGVHD. Bonin et al. [34] reported 13 patients with steroid-refractory aGVHD. However, four patients (31%) died within a period of 28 days after the first MSC transfusion and were not included in this analysis. Lucchini et al. [35] described a multicenter study of 11 pediatric patients, of whom eight were treated for steroid-refractory aGVHD and available for meta-analysis. Pérez-Simon et al. [38] included 18 patients in their study, but only 10 developed steroid-refractory aGVHD. Sánchez-Guijo et al. [39] described sequential third-party MSC therapy for refractory aGVHD in 25 patients, but one patient’s response was not evaluable. Banc et al. [40] described 55 patients treated for steroid-refractory aGVHD, all of whom were available for the analysis. Of the included patients, 136 (45.2%) showed CR, and 69 (22.9%) patients showed PR or MR. Most studies examined MSC therapy for steroid-refractory aGVHD. Patients’ ages varied from 0.2 to 69 years. Sample sizes ranged from 6 to 75. The demographic information of these studies is listed in Table 1.

**Table 1:** Characteristics of included studies.

Reference (years)	Country	n	Median age(range), years	Donor type	Source of Stem Cells (n)	Underlying Disease (n)	Conditioning Regimen (n)	Dose of MSC (cells per kg bodyweight)	No. of MSC infusions(n)
**Blanc et al.(2008)** [40]	Sweden, Netherlands,Italy and Australia	55	22 (0.5–64)	MRD 19, MUD 25,MMD 6,UCB 5	BM 19, PBSC 30,BM + PBSC 1,CB 5	leukemia 33,lymphoma 1, MM 2, MDS 6, others 13	MAC 37,RIC 17	1.4 × 10⁶ (0.4–9)	one (27),two (22),three (4),four (1),five (1)
**Fang et al.(2007**) [28]	China	6	40 (22–49)	MRD 3,MUD 3	BM2, PBSC 4	leukemia 6	MAC 6	1.0 × 10^6^	one (2),five (1)
**Lucchini et al.(2010)** [35]	Italy	11	10 (4–15)	unrelated 9,related 2	BM 9,PSBC 1,CB 1	leukemia 8,others 3	MAC 9,RIC 2	1.2 × 10⁶(0.7–3.7)	one (4),two to five (7)
**Prasad et al.(2011)** [29]	USA	12	6 (0.5–15)	unrelated 10,related 2	BM 2, PBSC 1, CB7, BM + CB 1, PBSC + CB 1	leukemia 6,others 6	MAC 11, RIC 1	2 × 10⁶, 8 × 10⁶	one to ten (7),more than ten (5)
**Kurtzberg et al.(2014)** [36]	USA, Canada, England, Italy, Finland, New Zealand, and Australia	75	7.8 (0.2–17.5)	unrelated 64,related 11	BM 25,PBSC 16,CB 28	leukemia 35,lymphoma 1, MDS 7, others 32	NA	2 × 10⁶	eight (35),twelve (40)
**Introna et al.(2014)** [30]	Italy	40	27.8 (1–65)	MRD 8,MUD 21,MMD 11	BM 15,PBSC 20,CB 5	malignant 36,nonmalignant 4	MAC 18,RIC 17,other 5	1.5 × 10⁶	more than two (40)
**Ringdén et al.(2006)** [31]	Sweden	8	56 (8–61)	MRD 4,MUD 3,MMD 1	BM 0,PBSC 1,CB 1	leukemia 6, MM 1, others 2	MAC 5,RIC 3,	1.0 × 10⁶ (0.7–9)	one (6),two (3)
**Pérez-Simon et al.(2011)** [38]	Spain	18	44 (21–66)	MUD 7,MRD 4,MMD 7	BM 5,PBSC 13	NA	MAC 7,RIC 11	2 × 10⁶ (0.3–3.7)	one (5),two (7),three (1),four (4)
**Bal et al.(2013)** [34]	the Netherlands and Italy	37	7 (0.7–18)	MRD 8,MUD 27,MMD 2	BM 18,PBSC 7,CB 12	leukemia 21, MDS 7, others 9	TBI-based 9, chemotherapy-based 28	1–2 × 10⁶	one to two (22),three to five (12), more than six (6)
**Bonin et al.(2009)** [32]	Germany	13	58 (21–69)	unrelated 9,related 4	PBSC 13	leukemia 5,lymphoma 2, MM 3,MDS 1,others 2	MAC 3, RIC 10	0.9 × 10⁶ (0.6–1.1)	one (1),two (8),three (2),four (1),five (1)
**Herrmann et al. (2012**) [33]	Australia	19	50 (21–61)	MRD 5,MUD 14	PBSC 19	leukemia 14,lymphoma 2,MDS 2, others 1	MAC 14,RIC 5	1.7–2.3 × 10⁶	two (10),three (1),more than three (8)
**Muroi et al.(2013)** [37]	Japan	14	52 (4–62)	related 2,unrelated 12	BM 9,PBMC 1,CB 4	leukemia 8, lymphoma 1,MDS 3, MM 1	MAC 11,RIC 3	2 × 10⁶	three (1),five (1),seven (1),eight (6),more than ten (5)
**Sánchez-Guijo et al. (2014)** [39]	Spain	25	NA (20–65)	NA	NA	leukemia 6,MDS 7, others 12	MAC 6,RIC 19	1.1 × 10⁶	two (4),three (3),four (18)

**Abbreviations**: MRD = matched related donor; MUD = matched unrelated donor; MMD = mismatched donor; UCB = unrelated cord blood; BM = bone marrow; PBSC = peripheral blood stem cells; CB = cord blood; MM = multiple myeloma; MDS = myelodysplastic syndrome; NA = not available; MAC = myeloablative conditioning; RIC = reduced-intensity conditioning; TBI = total body irradiation.

### MSC preparation and administration

Six studies used unrelated MSCs [29, 30, 32, 35, 36, 39], seven used both related and unrelated MSCs [28, 31, 33, 34, 37, 38, 40]. Ten used MSCs from mismatched donors [28–30, 32–37, 39], and three used both matched and mismatched cells [31, 38, 40]. Nine of the 12 studies cultured the MSCs in fetal bovine serum [28, 29, 31, 33–37, 40], one in human serum [38], and three in human platelet lysate [30, 32, 37]. Six of the 12 studies cryopreserved MSCs prior to administration [29, 30, 35–37, 39] and four used both fresh and cryopreserved MSCs [32–34, 40]. One study used only fresh MSCs [28], while the remainder of studies did not report this factor [31, 38]. Five studies reported the viability of prepared MSCs (range 70%–95%, median 80%) [29, 30, 33, 35, 40]. Twelve of the 13 studies derived MSCs from bone marrow, and one study used MSCs derived from adipose [28].

At present, no standard harvesting passages of MSCs are available. Therefore different harvesting passages were reported in the included studies. Eight studies reported the culture passage at MSCs harvest [31–37, 39, 40]. Only MSCs after first to fifth passage were used for therapy as reported by the aforementioned studies. Two studies administered MSCs in first to fourth passage [31, 40], two studies used MSCs in twice to third passage [33,34], one adopted MSCs in first to twice passage[32], one adopted MSCs in first to third passage[39], one study used MScs only in the third passage [35] and one administered MSCs only in the fifth passage [36]. Besides, data regarding the effect of harvesting passage on response were not available in most included studies. Studies have demonstrated that long-term passage affected the morphology and proliferation of hMSCs [41]. However, only first to fifth passages (referred to as early passage in some study [42]) were used for therapy, which minimized the effect of harvesting passage on response.

All the included studies described criteria for release of MSCs for clinical use. All of them stated that the cells were cultured negative for bacteria, mycoplasma and fungi before infusion. No homogeneous surface antigens of MSCs were identified by different studies. However, most studies identified MSCs as positive for surface antigens CD105 (SH-2) [28–37, 39, 40], CD 73 (SH-3, SH-4) [29–40], CD90 [29, 30, 32–40] and negative for markers of hematopoietic lineages CD14 [28–36, 39, 40], CD34 [28–40], and CD45 [36, 37, 39].

### Toxicity

No infusion-related toxicity was observed during or immediately after the administration of MSC in most studies [28, 30–35, 37, 38, 40]. Only three studies reported possible toxicity related to infusion of MSCs [29, 36, 39]. Prasad et al [29] reported one patient transferred to the intensive care unit within 24 hours of the second hMSC infusion and the other patient with calcified ectopic lesions developed in the scalp and foot after hMSC therapy. Sánchez-Guijo [39] et al described one patient who developed a cardiac ischemic event 24 hours after the first infusion. However, those events were deemed not likely related to the hMSC infusion. Kurtzberg et al [36] mentioned one patient with infusion-related toxicity which resolved without sequelae and toxicity in 6 patients which were considered possibly related to MSCs infusion.

### FCS versus platelet

No significant association was detected when comparing fetal bovine serum with human platelet lysate in the recipients’ clinical responses (CR: 0.58±0.21 VS 0.29±0.17, p>0.05; ORR: 0.74±0.19 VS 0.74±0.03, p>0.05) under two independent sample t-test.

### Children vs. adults

**Fig 2:**
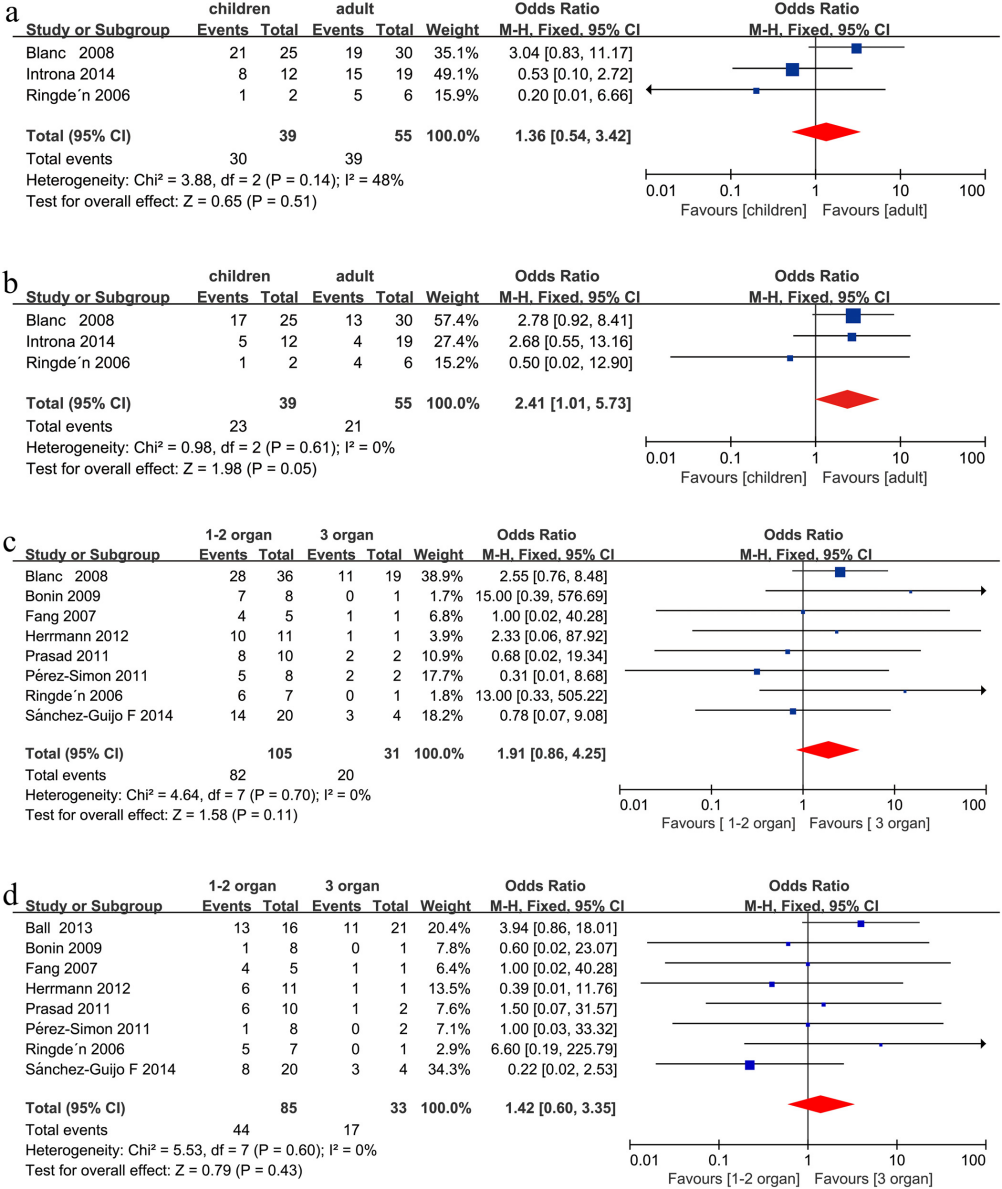
(a) Meta-analysis of the overall response (ORR) of children vs. adults after mesenchymal stem cell (MSC) infusion. (b) Meta-analysis of the complete response (CR) of children vs. adults after MSC infusion. (c) Meta-analysis of the ORR with one or two involved organs vs. three involved organs after MSC infusion. (d) Meta-analysis of the CR with one or two involved organs vs. three involved organs after MSC infusion.

There was a trend towards a better CR in children compared with adults after MSC infusion in steroid-refractory aGVHD (OR = 2.41, 95%CI: 1.01–5.73, p = 0.05, I^2^ = 0%, Fig 2b). No significant difference was found between the two groups in ORR (OR = 1.36, 95%CI: 0.54–3.42, p = 0.51, I^2^ = 48%, Fig 2a) after MSC infusion in steroid-refractory aGVHD under a fixed effect model.

### Fewer involved organs vs. more involved organs

**Fig 3:**
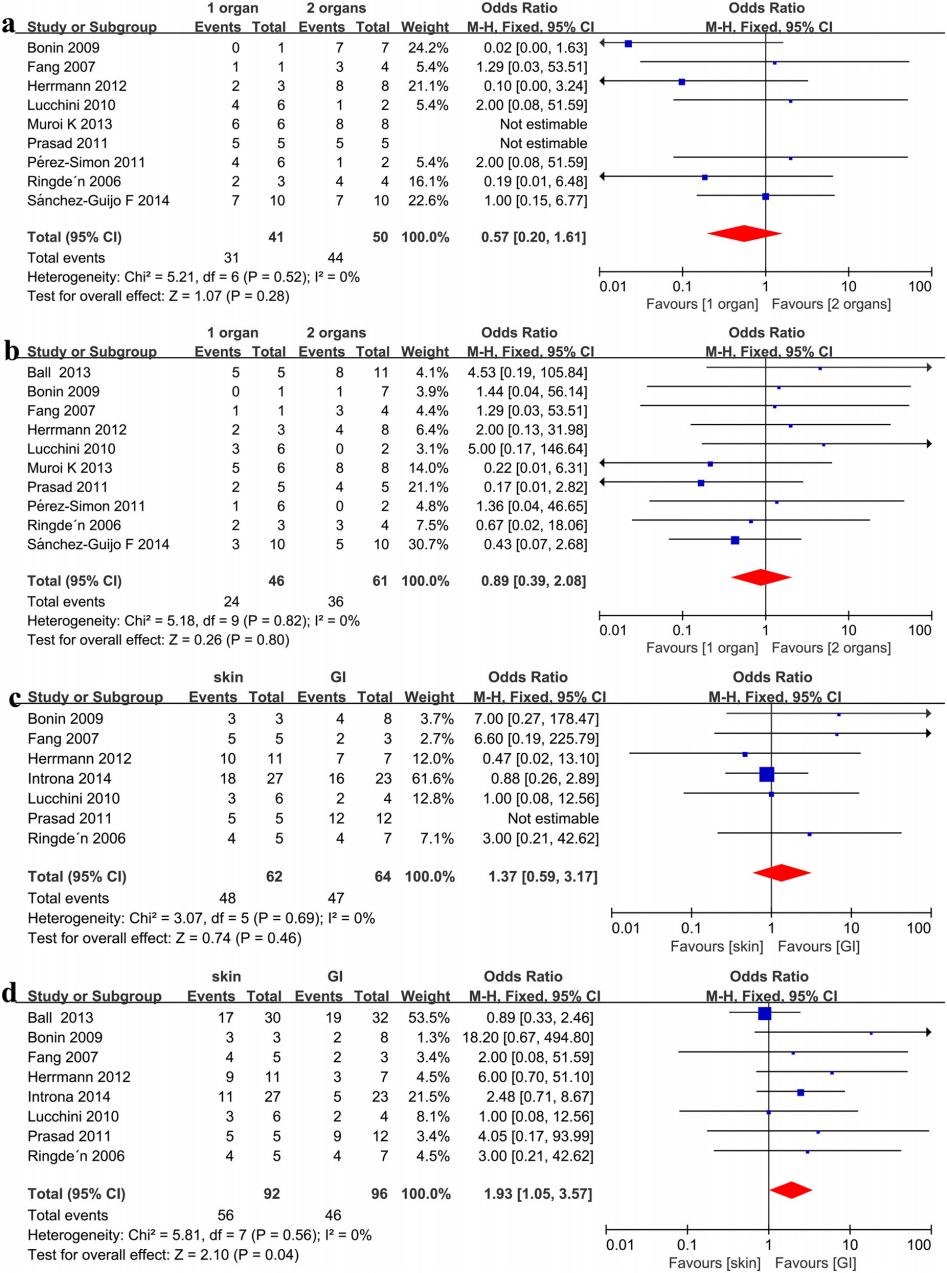
(a) Meta-analysis of the overall response (ORR) with one involved organ vs. two involved organs after MSC infusion. (b) Meta-analysis of the complete response (CR) with one organ vs. two involved organs after MSC infusion. (c) Meta-analysis of the ORR of skin vs. gastrointestinal (GI) involvement after mesenchymal stem cell (MSC) infusion. (d) Meta-analysis of the CR of skin vs. GI involvement after MSC infusion.

No significant association was detected when comparing one or two involved organs with three involved organs in the recipients’ clinical responses (ORR: OR = 1.91, 95%CI: 0.86–4.25, p = 0.11, I^2^ = 0%, Fig 2c; CR: OR = 1.42, 95%CI: 0.60–3.35, p = 0.43, I^2^ = 0%, Fig 2d) under a fixed effect model after MSC infusion in steroid-refractory aGVHD. No significant association was detected when comparing one involved organs with two involved organs in the recipients’ clinical responses (ORR: OR = 0.57, 95%CI: 0.20–1.61, p = 0.28, I^2^ = 0%, Fig 3a; CR: OR = 0.89, 95%CI: 0.39–2.08, p = 0.80, I^2^ = 0%, Fig 3b) under a fixed effect model after MSC infusion in steroid-refractory aGVHD.

### Skin vs. GI vs. liver

**Fig 4:**
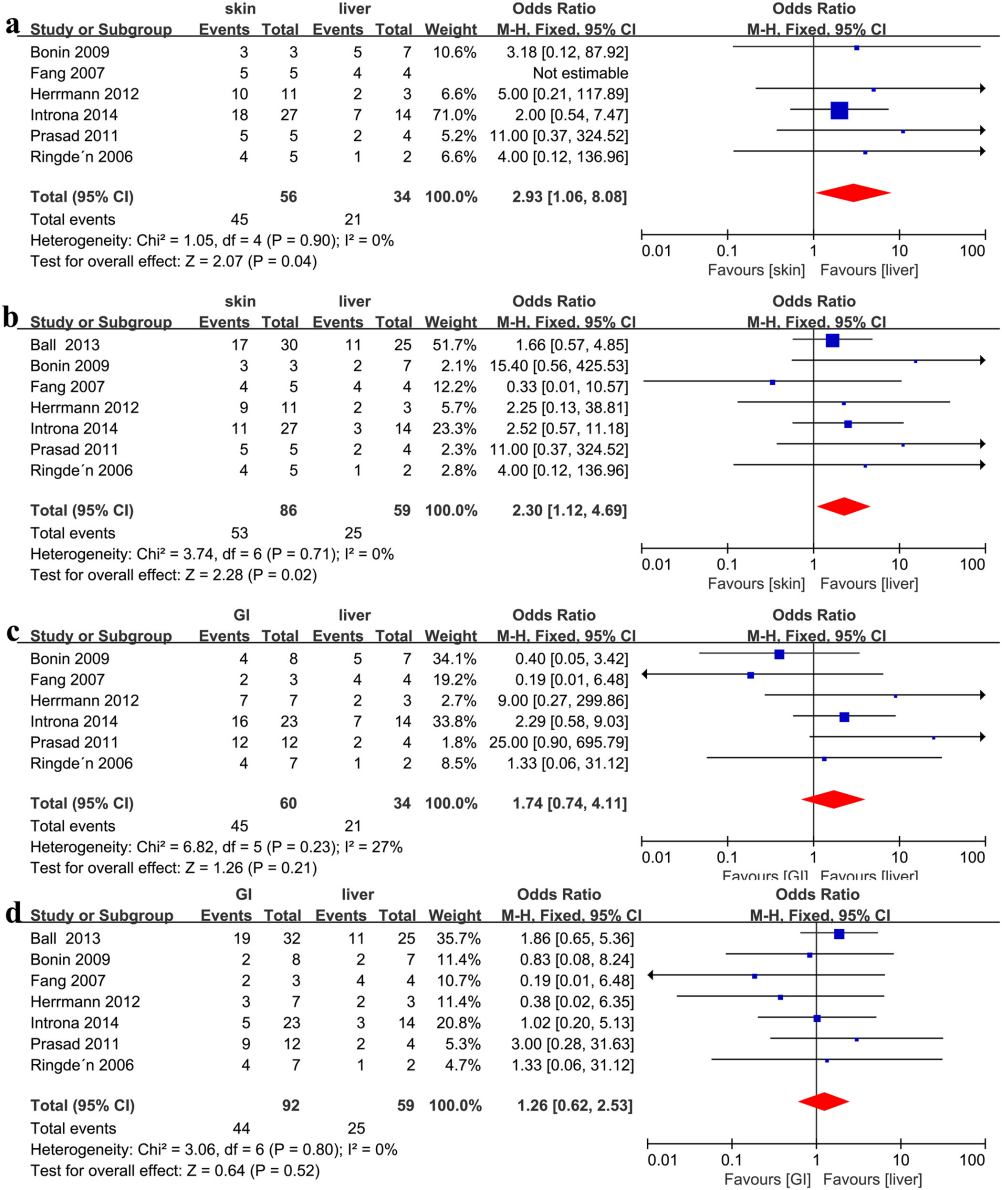
(a) Meta-analysis of the overall response (ORR) of skin vs. liver involvement after MSC infusion. (b) Meta-analysis of the complete response (CR) of skin vs. liver involvement after MSC infusion. (c) Meta-analysis of the ORR of gastrointestinal (GI) vs. liver involvement after mesenchymal stem cell (MSC) infusion. (d) Meta-analysis of the CR of GI vs. liver involvement after MSC infusion.

Recipients with skin steroid-refractory aGVHD showed a better CR compared with GI steroid-refractory aGVHD (OR = 1.93, 95%CI: 1.05–3.57, p = 0.04, I^2^ = 0%, Fig 3d), but exhibited a similar ORR (OR = 1.37, 95% CI: 0.59–3.17, p = 0.46, I^2^ = 0%, Fig 3c). Recipients with skin steroid-refractory aGVHD showed a better clinical response than patients with liver (ORR: OR = 2.93, 95%CI: 1.06–8.08, p = 0.04, I^2^ = 0%, Fig 4a, and CR: OR = 2.30, 95%CI: 1.12–4.69, p = 0.02, I^2^ = 0% Fig 4b, respectively) steroid-refractory aGVHD. Patients with GI and liver steroid-refractory aGVHD exhibited a similar clinical response after MSC therapy (ORR: OR = 1.74, 95%CI: 0.74–4.11, p = 0.21, I^2^ = 27%, Fig 4c, and CR: OR = 1.26, 95%CI: 0.62–2.53, p = 0.52, I^2^ = 0%, Fig 4d, respectively).

### Grade II vs. Grade III–IV

**Fig 5:**
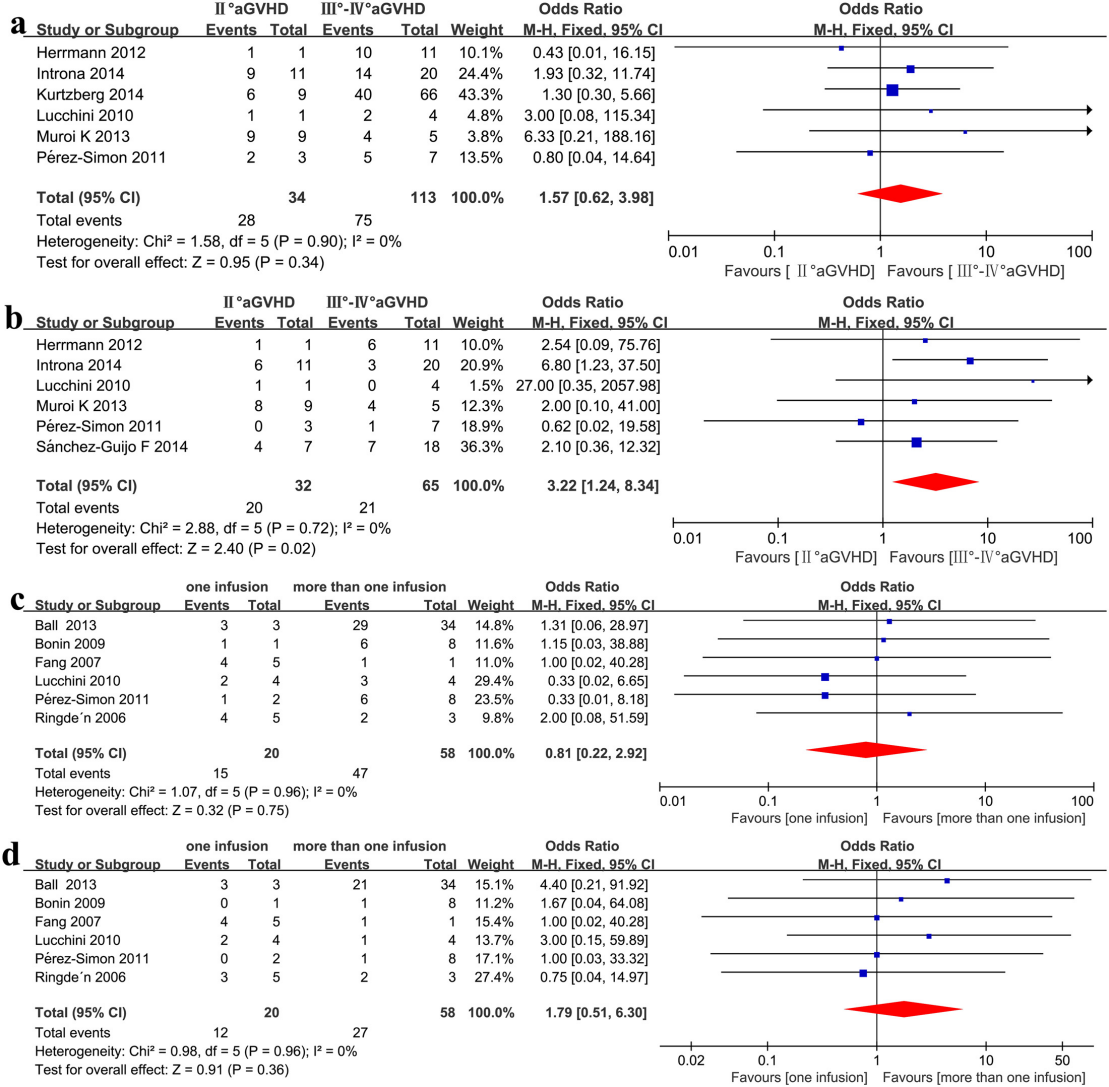
(a) Meta-analysis of the overall response (ORR) of grade II vs. grade III–IV aGVHD after MSC infusion. (b) Meta-analysis of the complete response (CR) of grade II vs. grade III–IV aGVHD after MSC infusion. (c) Meta-analysis of the overall response after one infusion vs. more than one infusion. (d) Meta-analysis of the complete response after one infusion vs. more than one infusion.

Patients with grade II steroid-refractory aGVHD showed a better CR compared with recipients with grade III–IV steroid-refractory aGVHD (OR = 3.22, 95%CI: 1.24–8.34, p = 0.02, I^2^ = 0%, Fig 5b), but exhibited a similar ORR (OR = 1.57, 95%CI: 0.62–3.98, p = 0.34, I^2^ = 0%, Fig 5a).

### One infusion vs. more than one infusion

No significant association was detected when comparing one infusion with more than one infusion in the recipients’ clinical responses (ORR: OR = 0.81, 95%CI: 0.22–2.92, p = 0.75, I2 = 0%, Fig 5c; CR: OR = 1.79, 95%CI: 0.51–6.30, p = 0.36, I2 = 0%, Fig 5d) under a fixed effect model in steroid-refractory aGVHD.

### Induction vs. completion therapy

**Fig 6:**
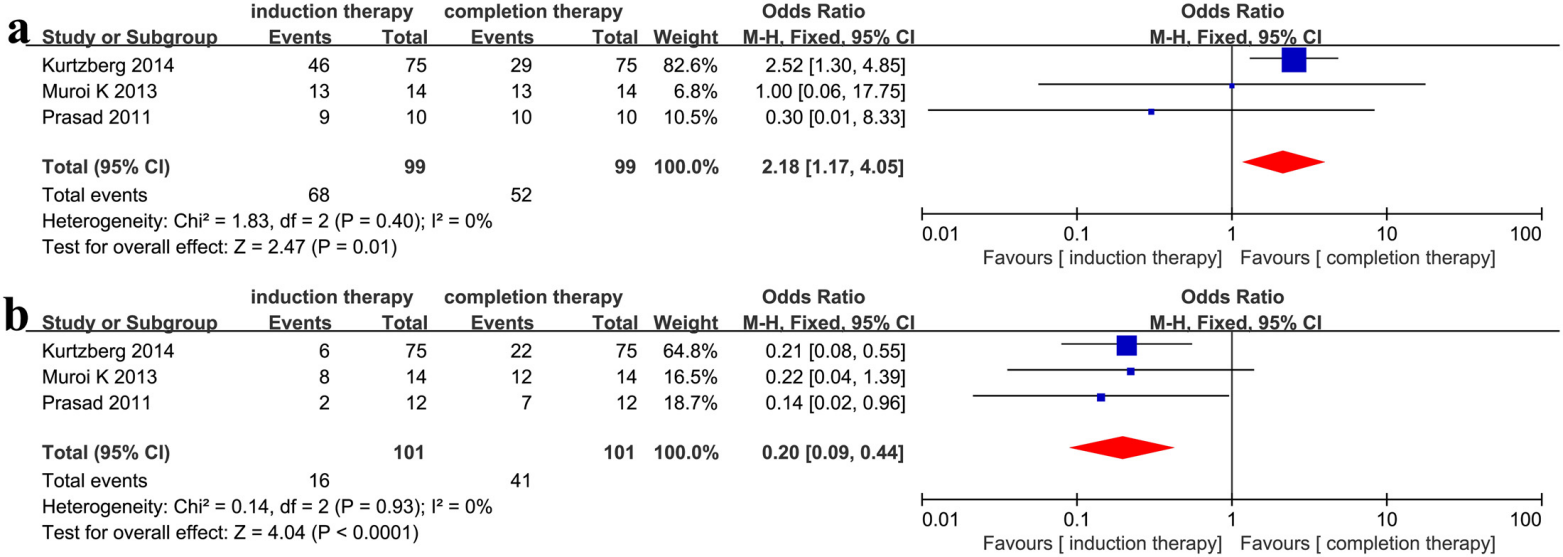
(a) Meta-analysis of the overall response (ORR) in induction therapy vs. completion therapy after MSC infusion. (b) Meta-analysis of the complete response (CR) in induction therapy vs. completion therapy after MSC infusion.

Initial responses after the first few cycles and after completion of MSC therapy were recorded. The CR upon completion therapy improved compared with the initial treatment (OR = 0.20, 95%CI: 0.09–0.44, p < 0.0001, I^2^ = 0%, Fig 6b) though the ORR declined (OR = 2.18, 95%CI: 1.17–4.05, p = 0.01, I^2^ = 0%, Fig 6a). This may indicate that increasing the time course of MSC infusions may improve CR but reduce ORR.

### Publication bias

The results of Begg’s and Egger’s tests did not suggest any evidence of publication bias (Begg’s rank correction test and Egger’s regression test p values > 0.05).

## Discussion

This systematic review, which compiled all trials regarding factors affecting the efficacy of MSC treatment in steroid-refractory aGVHD, yielded several clinically significant factors: skin involvement and lower aGVHD grade were associated with a better clinical response. Completion therapy can achieve better CR though the ORR declined. There was also a trend towards a better clinical response in children compared with adults. No infusion-related toxicity was observed during or immediately after the administration of MSC in most studies, indicating that MSCs were safe treatment for steroid-refractory aGVHD. These data are important for physicians considering MSCs for patients with steroid-refractory aGVHD.

The treatment of severe aGVHD continues to be very challenging. The current therapies do not offer significant benefits, and no therapy has been Federal Drug Administration-approved for the treatment of aGVHD. In general, approximately 50% of all patients with aGVHD initially respond to first-line steroid therapy. However, the response rates are still low and lot of people require further therapeutic intervention. Among the most recent therapeutic approaches for refractory aGVHD, MSCs hold a key position; however, studies concerning factors affecting the efficacy of MSC treatment in steroid-refractory aGVHD show controversial results.

In this meta-analysis, we found that age may play an important role in MSC therapy efficacy, as children tended to show a better clinical response than adults. This is supported by the findings of Zhuo et al. [43], who found that recipient age was an important contributor to the outcome of cell therapy. However, as the current results did not exhibit a significant difference, further studies to confirm this tendency are warranted. The current analysis also demonstrated that more patients with skin involvement responded favorably to MSC infusion, compared to patients with liver or GI involvement. The data provided by this meta-analysis also indicated that although patients’ ORRs were not different in terms of aGVHD severity, there was a higher degree of CR in lower grade aGVHD patients after MSC infusion, suggesting a better outcome for lower grade aGVHD after MSC treatment. In addition, there was a higher degree of CR in patients who completed therapy, suggesting that increasing the duration of infusions may improve patients’ CR when induction therapy is not satisfied. However, the ORR declined when MSCs were further infusions were done. Passages performed before harvesting MSC may have an impact on response. However, data regarding the effect of harvesting passage on response were not available in most included studies. No significant association was detected when comparing fetal bovine serum with human platelet lysate in the recipients’ clinical responses. Many studies [44, 45] suggested that platelet lysate was a suitable alternative to FBS for use in equine MSC expansion, which confirmed our conclusion. We found no differences in responses based on the number of organs involved. There was also no significant difference after MSC treatment when comparing GI and liver steroid-refractory aGVHD. Although our meta showed that no significant difference was found when comparing one infusion with more than one, and that complete therapy seemed to improve the CR but decrease ORR, these conclusions should be carefully made unless two aspects be considered: one, the studies included were too few; in addition, patients who received more than one infusion were likely to have more severe grade of aGVHD. For these patients equivalent outcome can be achieved if more than on infusion were given, indicating potential benefit of more than one infusion. The seeming decreased ORR may also disappear if more studies were included and patients were in the same condition before MSCs infusion. More prospective clinical trials are needed in the future.

At present, little is known about the mechanisms by which MSCs inhibit GVHD. In vitro, MSCs have varied effects on immune cells, including T cells, antigen-presenting cells, natural-killer cells, and B cells [46–48]. For inhibition of inflammation, MSCs may employ multiple mechanisms. The most studied and best understood potential mechanisms are direct cell-to-cell contact and paracrine regulation [49, 50]. As MSCs migrate to various tissues, the different number and proportion of migrated MSCs in different organs may possibly explain these phenomena. Although recent reports suggest a role for chemokines in human MSC migration [51], the signaling pathways responsible for their directed migration remain unknown.

Several limitations in our analysis merit consideration. First, the paucity of randomized controlled trials (RCTs) prompted us to incorporate non-randomized comparative trials to the meta-analysis. However, most comparisons were not statistically heterogeneous, which may support the hypothesis that non-randomized comparative studies did not introduce significant bias. Notwithstanding, considering meta-analysis of RCTs as the gold standard of evidence-based medicine, the robustness of our results may be hampered by the lack of RCTs. Second, the power of our analysis is limited by the small number of patients in each comparison. Third, no standard harvesting passages of MSCs were available at present, the difference of which may have an influence on patients’ response. However, only first to fifth passages were used for therapy, which minimized the effect of harvesting passage on response. Fourth, patients of steroid refractory aGVHD receive other drugs before and during MSCs infusions, which may also had an impact on patient response. The tapering or increasing of conventional immunosuppressive therapy after MSC administration was left to the clinical judgment of the treating teams in most studies, therefore conventional immunosuppressive therapy was not homogenous and not able to be analyst. Besides, most studies did not discuss the effect of other drug on MSCs efficacy.

Our systematic review and meta-analysis shows that MSC therapy may have the best efficacy in patients with lower grade aGVHD and only skin involvement. When induction therapy does not achieve satisfactory results, continuing therapy may be helpful for some patients. Children tended to show better complete response than adults; this should be further investigated in more prospective randomized trials. Additional well-controlled randomized trials that further assess the feasibility of MSCs in different settings of hematological transplantations are needed.

## Supporting Information

S1 PRISMA Checklist (DOC)
